# Nasal Drug Delivery of Anticancer Drugs for the Treatment of Glioblastoma: Preclinical and Clinical Trials

**DOI:** 10.3390/molecules24234312

**Published:** 2019-11-26

**Authors:** Franciele Aline Bruinsmann, Gustavo Richter Vaz, Aline de Cristo Soares Alves, Tanira Aguirre, Adriana Raffin Pohlmann, Silvia Stanisçuaski Guterres, Fabio Sonvico

**Affiliations:** 1 Programa de Pós-Graduação em Ciências Farmacêuticas, Universidade Federal do Rio Grande do Sul, Porto Alegre 90610-000, Brazil; fbruinsmann@gmail.com (F.A.B.); alves.alinecs@yahoo.com.br (A.d.C.S.A.); adriana.pohlmann@ufrgs.br (A.R.P.); silvia.guterres@ufrgs.br (S.S.G.); 2 Food and Drug Department, University of Parma, Parco Area delle Scienze 27/a, 43124 Parma, Italy; richtervaz@gmail.com; 3 Laboratório de Nanotecnologia Aplicada à Saúde, Programa de Pós-Graduação em Ciências da Saúde, Universidade Federal do Rio Grande, Rio Grande, RS 96210-900, Brazil; 4 Programa de Pós-Graduação em Biociências, Universidade Federal de Ciências da Saúde de Porto Alegre, Porto Alegre, RS 900500-170, Brazil; tanira@ufcspa.edu.br; 5 Departamento de Química Orgânica, Instituto de Química, Universidade Federal do Rio Grande do Sul, Porto Alegre 91501-970, Brazil

**Keywords:** nasal delivery, glioblastoma multiforme, drug delivery, nanoparticles, nose-to-brain delivery, pre-clinical studies, clinical evaluation

## Abstract

Glioblastoma (GBM) is the most lethal form of brain tumor, being characterized by the rapid growth and invasion of the surrounding tissue. The current standard treatment for glioblastoma is surgery, followed by radiotherapy and concurrent chemotherapy, typically with temozolomide. Although extensive research has been carried out over the past years to develop a more effective therapeutic strategy for the treatment of GBM, efforts have not provided major improvements in terms of the overall survival of patients. Consequently, new therapeutic approaches are urgently needed. Overcoming the blood–brain barrier (BBB) is a major challenge in the development of therapies for central nervous system (CNS) disorders. In this context, the intranasal route of drug administration has been proposed as a non-invasive alternative route for directly targeting the CNS. This route of drug administration bypasses the BBB and reduces the systemic side effects. Recently, several formulations have been developed for further enhancing nose-to-brain transport, mainly with the use of nano-sized and nanostructured drug delivery systems. The focus of this review is to provide an overview of the strategies that have been developed for delivering anticancer compounds for the treatment of GBM while using nasal administration. In particular, the specific properties of nanomedicines proposed for nose-to-brain delivery will be critically evaluated. The preclinical and clinical data considered supporting the idea that nasal delivery of anticancer drugs may represent a breakthrough advancement in the fight against GBM.

## 1. Introduction

Malignant brain tumors are devastating diseases with high morbidity and mortality in adults. In children, they represent the second leading cause of cancer related deaths [1,2]. Glioblastoma multiforme (GBM) is the most common and the most lethal malignant primary brain tumor in adults. Moreover, GBM shows a recurrence rate of higher than 90%, even after multimodal treatment that combines surgery and chemotherapy [3]. The World Health Organization (WHO) classifies GBM as Grade IV, which is the highest in CNS tumors classification, based on the level of malignancy. Indeed, GBM is the most invasive and aggressive type of glial tumors. Primary GBMs, i.e., which arise without a known precursor, are the most common form of GBM (~90%), and they tend to be more aggressive and generally affect older patients. Alternatively, secondary GBMs develop slowly through progression from a lower-grade astrocytic tumor (WHO Grade II or III). These tumors are more frequent in younger patients and they are characterized by a significantly more favorable prognosis. Histologically, primary and secondary GBMs are indistinguishable, but they differ in their genetic and epigenetic profiles [4,5]. It is estimated that GBM has an incidence of 3.21 cases per 100,000 persons in the United States [6], with median survival time of around 7–15 months from the time of diagnosis [7]. The incidence rates of glioblastoma increase with age, with the highest rates in individuals aged between 75 and 84 years [6]. Patient survival at five years form diagnosis is lower than 5% [8]. The etiology of GBM is complex and it has not been fully elucidated, but a mix of genetic and environmental factors is the most likely cause of the disease [9].

GBM is a tumor that is extremely challenging to treat due to its highly invasive nature. Current treatments are based on maximal surgical resection, followed by radiotherapy and adjuvant chemotherapy [10]. Temozolomide (TMZ) is the standard chemotherapeutic agent for the treatment of GBM. This second-generation imidazotetrazinone derivative exerts its anticancer effect through DNA methylation [11]. TMZ is, in fact, a prodrug, which is spontaneously hydrolyzed at physiological pH to its alkylating metabolite 3-methyl-(triazen-1-yl)-imidazole-4-carboxamide (MTIC) [11]. TMZ is orally administered and it leads to fewer side effects when compared with other chemotherapeutic agents parenterally administered [12]. Notwithstanding, its clinical effectiveness remains limited, since tumors rapidly develop resistance to the treatment [13]. In addition, several GBM cases are intrinsically resistant to TMZ, even during initial treatments. This inherent resistance is a consequence of various defense mechanisms, such as the expression of multi-drug resistance proteins and DNA repair systems impairment [14]. The extensive and complete surgical resection of GBM represents the most effective way to increase the survival of GBM patients [15]. However, the surgical intervention is difficult and, in most cases, less than 90% of the tumor can be removed [16]. In fact, these tumors exhibit a high degree of invasiveness and are often localized in important functional areas of the brain, including areas that are involved in the control of speech, motor functions, and senses [17]. Furthermore, the blood–brain barrier (BBB) limits the passage of molecules, including many anticancer drugs, from the bloodstream into the brain [18]. As a consequence of the restrictive nature of the BBB combined with the low brain permeability to most drugs, high doses have to be administered to obtain therapeutic concentrations in the brain. Although several strategies have been proposed to overcome these obstacles (e.g., oncolytic viruses, targeted therapies, immunotherapy, vaccines, etc.), brain delivery of therapeutic molecules against glioblastoma remains a challenge [19].

**Figure 1:**
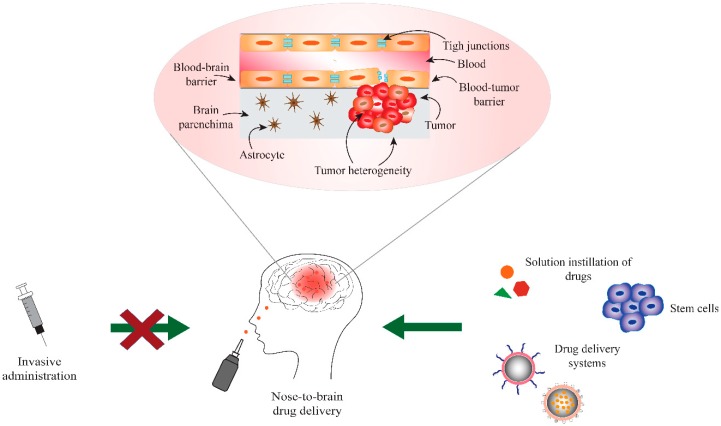
Obstacles and opportunities in the nose to brain drug delivery approaches for the treatment of glioblastoma multiforme (GBM).

Nose-to-brain delivery has been proposed as a non-invasive approach to directly access the brain through bypassing the BBB. It is actively investigated as an alternative administration route for the delivery of pharmaceutically active molecules that are potentially useful in a number of CNS disorders. In particular, the intranasal delivery route could represent a major breakthrough in the treatment of GBM, since it offers an effective drug delivery approach for a number of innovative therapeutic strategies (Figure 1).

## 2. Blood-Brain and Blood-Tumor Barriers (BBB/BTB)

The BBB protects the CNS by exhibiting a highly selective permeability to substances that are present in blood in order to preserve brain homeostasis and ensure the correct neuronal functioning of the brain. The BBB is a cellular barrier and its properties are mainly due to the presence of tight junctions (TJ) between the endothelial cells of brain capillaries and the involvement of surrounding cells, i.e., adjoining pericytes, astrocytes, and microglia [20,21]. The BBB selectively ensures the supply of essential nutrients and oxygen into the CNS, while also preventing the passage of macromolecules as well as of undesirable toxic or infectious agents, thus providing an adequate brain homeostasis [22,23]. Along with these defensive functions, the BBB also prevents the entry of xenobiotic drugs from the blood into the brain. The BBB is only normally permeable to small and lipophilic molecules, with a molecular weight (Mw) lower than 400–500 Da [24]. In addition, the LogP that is required for an efficient transport across the BBB is estimated to be in the range of 1.5 and 2.7 [25]. Low hydrogen-bonding potential is another important drug characteristic to ensure access to the CNS [26]. 

In brain tumors, the microenviroment differs from that of the healthy brain, since the morphology, function, and organization of BBB are affected. The result of this alteration is the formation of the so-called blood-tumor barrier (BTB) [27,28,29]. In high-grade gliomas, as in glioblastoma, the BTB is made from existing and newly formed blood vessels that contribute to the delivery of nutrients and oxygen to the tumor and facilitate glioma cell migration to other parts of the brain [27,30]. The tumor expansion creates hypoxic areas that trigger the overexpression of vascular endothelial growth factor (VEGF) and, consequently, the promotion of neoangiogenesis [31]. The neovascularization process commonly leads to the formation of abnormal vessels that are then able to sustain the high metabolic activity of the tumor cells. The increased fenestration or loss of tight junctions between endothelial cells characterizes these vessels. Furthermore, these new vascular endothelial cells are overexpressing caveolae, have increased pinocytotic activity, and are rich in mitochondria [31,32,33]. Nevertheless, the BTB presents continuous fenestrated vessels with a defined pore size, which precludes the entrance of hydrophilic compounds and large molecules to the brain tumor [30,34]. As a consequence, most of the antitumor agents are not delivered to the brain tumors due to the presence of BTB [30,34]. The permeability of the BTB can increase as the brain tumor progresses to the late development stages, since an impairment of the BBB/BTB often occurs along with an intensification of the enhanced permeability and retention (EPR) effect, which results in a tendency of large molecules and particles in the nanoscale to accumulate at the brain tumor site [32,34].

## 3. Nose-to-Brain Drug Delivery

The choice of the treatment and, ultimately, average patient survival, depend on the glioma type, size, location, and grade [35,36]. In some cases, the median survival can be extended by the addition of adjuvant chemotherapy (TMZ) to the radiotherapy (RT) [37,38]. Stupp et al. found that the median survival of patients receiving TMZ in addition to RT was 14.6 months when compared with 12.1 months among those who were assigned to RT alone [39]. However, despite the benefits, treatment with TMZ can entail some severe side effects, such as nausea, vomiting, lymphopenia, neutropenia, thrombocytopenia, fatigue, disturbed sleep, and depression [40,41,42]. Furthermore, the risk of neurocognitive impairment is increased when RT is administered to the whole brain and even more when the chemotherapy is associated to RT [43,44,45].

Approaches to overcome the physiological barriers and limitations to access the human CNS include the exploitation of ways that are suitable for the direct administration of the drug to the brain. This can be done by intraventricular, intrathecal, or nasal administration [46]. The intrathecal administration requires some risky surgical procedures and the drugs administered can present limited distribution throughout the brain parenchyma and cerebrospinal fluid (CSF). A better distribution into the CSF can be obtained by intraventricular administration, but this type of administration requires the implant of drug release controlling reservoirs. Furthermore, some severe side effects can occur when drugs are intrathecally and intraventricularly administrated, including meningitis, arachnoiditis, and focal neurologic injury. Moreover, these approaches will result in the potential increase of drug-related toxicities because of the restricted volume of distribution if CSF flow abnormalities are present [47,48]. As a consequence, these invasive brain administration approaches appear to only be applicable to a limited number of selected patients.

The intranasal route of administration is able to bypass the BBB and it appears to be an alternative route for the delivery of drugs to the CNS. In fact, several evidences have been provided in the scientific literature supporting the claim that drugs can reach the CNS after administration into the nasal cavity [49,50,51]. It is essential to understand the mechanisms of transport of the compounds, the anatomy of the nervous system, and the pathophysiology of the disease, as well as several experimental parameters, in order to design a formulation that allows access to the CNS through the intranasal route efficiently [52]. In the next paragraphs, the anatomical organization of the nasal cavity will be briefly discussed, focusing on the structures that are necessary for understanding nose-to-brain transport, since excellent descriptions can be found in many review papers and textbooks [53]. 

Anatomically, the nose presents two cavities limited by a septal and a lateral wall dominated by the turbinates, structures that are responsible for the temperature regulation and humidification of the inspired air [54]. The innervation of the human nasal cavity can be divided into sensory and olfactory nerves. The sensory innervation consists of the first and second divisions of the trigeminal nerve (ophthalmic nerve and maxillary nerve), while the olfactory innervation is ascribed to the olfactory nerve [55,56]. The nasal cavity can be divided into three regions: the vestibular region, the respiratory region, and the olfactory region. The vestibular region, which is located in the frontal part of the nasal cavity, is followed by the respiratory region that presents approximately 130 cm^2^ of area, and it is characterized by the sensory/trigeminal innervation. In humans, this is the largest region and it can reach up to 80–90% of the nasal cavity [57]. The respiratory epithelium is responsible for covering the nasal conchae, i.e., bone projections of the lateral walls of the nasal cavity and also the paranasal sinuses, i.e., cavities in the facial bones that communicate with the nasal cavity [58]. The last region of the nasal cavity is the olfactory region, which, in humans, represents approximately 10% of the nasal cavity surface area. This region is located in the upper part of the nasal fossa, below the lamina cribrosa (or cribriform plate) of the ethmoid bone and it is innervated by the olfactory nerve [54,59,60]. Olfactory cells are bipolar unmyelinated neurons that present dendrites with terminations protruding above the surface of the nasal mucosa interspaced between the supporting cells and an axon extending through the connective tissue towards the olfactory bulb located in the CNS [61]. The constant replacement of olfactory receptor neurons makes the olfactory mucosa relatively “leaky”, allowing for the nose-to-brain transport [62]. The molecular weight and the hydrophilic/lipophilic nature of the drug directly influence the absorption of the drugs through the nasal route. Poor bioavailability after nasal administration is generally observed for drugs with a molecular weight greater than 1 kDa [63]. Lipophilic compounds presenting a molecular weight lower than 1 kDa in some cases present bioavailability close to 100%, i.e., similar to drug exposure obtained after intravenous administration [64]. 

**Figure 2:**
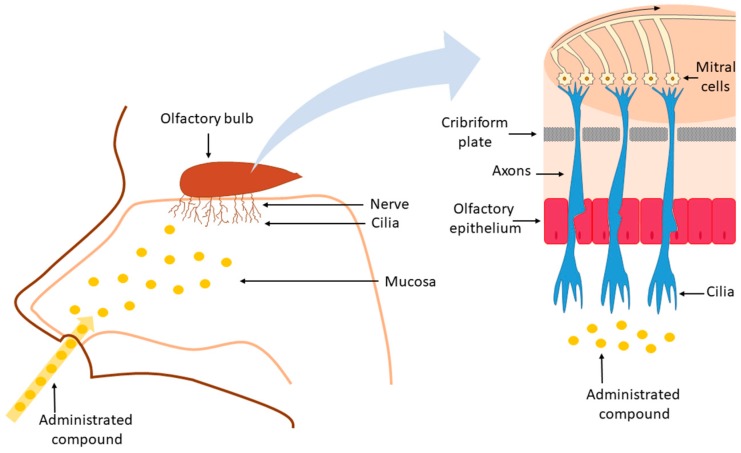
Structures involved in nose-to-brain transport by the olfactory pathway.

Mucociliary clearance takes place after the administration into the nasal cavity of a formulation, generally starting from the vestibular region [65]. The formulation is then moved to the posterior region of the nasal cavity in the direction of the respiratory and olfactory regions. The transport to the brain of the drug or of the formulation itself can happen via five different pathways: the olfactory nerve, the trigeminal nerve, the lymphatic, the CSF, and the vascular pathway. The nose-to-brain transport can occur via a single route or through a combination of pathways mentioned above, depending of the nature of the drug, the characteristics of the formulation and the physiological conditions [66]. The substances can then move towards lamina propria and the brain by two different mechanisms, the intracellular and the extracellular transport mechanisms. Once at the lamina propria, the substances follow the perineural channels that are created by a glial cell type, the olfactory ensheathing cells, which cover the non-myelinated axons, cross the cribriform plate, and enter into the CSF and olfactory bulb (Figure 2).

From the CSF, the substances are then distributed throughout the brain via bulk flow after being mixed with the interstitial fluid. They are also rapidly distributed throughout the CNS via perivascular transport [52,67]. 

The olfactory nerve pathway exploits the unique position of olfactory receptor neurons located in the olfactory mucosa, being a direct connection between CNS and the external environment [23,68]. The permeation of the compounds to the CNS occurs along or within the neurons present at the olfactory epithelium through the olfactory pathway [69]. In the case of intraneuronal axonal transport, the compounds are internalized in the olfactory epithelial neurons and then conveyed to the olfactory bulb, thus enabling the compounds to be further distributed to the rest of the brain [70]. In the intracellular form of transport, generally preferred by substances with a hydrodynamic radius above 20 nm, permeation can happen through endocytosis by sensory olfactory neurons and subsequent intraneuronal transport to the olfactory bulb, or through transcellular transport to the cells of the lamina propria [49,71]. However, the intraneuronal axonal transport is usually quite slow, with the times of delivery to the CNS ranging from hours to several days. The paracellular transport involves the absorption of the compounds across the nasal epithelium and the extracellular diffusion associated with bundles of nerves with consequent migration to the cranial compartment [72]. In fact, the perineural spaces of the cranial nerves, as in the case of the olfactory and trigeminal nerves, seem to allow for communication with the CSF of the subarachnoid space, which allows a rapid access route for the molecules absorbed across the nasal mucosa to reach the CNS [70]. The direct nose-to-brain transport can also occur by the trigeminal nerve, which innervates the respiratory and olfactory epithelia of the nasal cavity and allows the access of compounds to the caudal and rostral sections of the brain after intranasal administration [73,74]. However, it is important to point out that it is not generally possible to determine a specific/exclusive way that molecules/peptides access the CNS after the nasal administration, because this access can occur simultaneously by multiple pathways [66]. In fact, in conjunction with these pathways, other mechanisms can also provide access to the CNS from the nasal cavity, such as the nasal and the brain lymphatic systems that connect to the CSF and CNS interstitial spaces drainage by bulk flow mechanisms through perivascular channels surrounding blood vessels [66,75]. Another mode for the penetration of compounds into the CNS from the nasal respiratory region is through an indirect way via the vascular pathway. In fact, nasal blood vessels present continuous but fenestrated endothelia that enable the small molecule passage and the delivery to the brain by distribution across the BBB [74,76].

Despite the numerous advantages, nose-to-brain drug delivery can be limited as a result of some aspects that are related to the intranasal administration, such as low bioavailability of peptides and proteins due to enzymatic degradation, high clearance from the nasal cavity due to mucociliary transport, and other restrictions determined by the anatomy of the nasal cavity, such as small administration volume, limited surface area of the olfactory mucosa, mucus barrier, etc. In terms of enzymatic degradation, it can occur in the lumen of the nasal cavity or during transit across the epithelial barriers due to the presence of exo-peptidases, such as mono- and diaminopeptidases, which can cleave peptides at their N and C terminal and endo-peptidases that can attack internal peptide bonds [77]. The mucus present in the upper respiratory region acts as a physical and chemical barrier that entraps particles and molecules. The mucus is then drained from the nasal cavity into the pharynx through ciliary movement to be swallowed or expectorated [58]. Despite these limitations, Quintana and collaborators [78] reported results from a clinical trial that investigated the intranasal delivery of oxytocin (OT) to 16 male health adults. The treatment groups were divided in: two different doses of intranasal OT, i.e., eight and 24 IU; one IU intravenous OT and placebo with a period of at least six days between treatments to prevent potential carryover and/or administration-related effects. The blood samples were collected to determine the peripheral levels of OT, cross-reactive vasopressin (AVP), and cortisol. All of the treatments produced similar plasma OT increases when compared with placebo. However, the behavioral data that were obtained by emotional expression evaluation suggested that OT delivered intranasally while using a Breath Powered bi-directional device (Optinose, Oslo, Norway) reached the brain and influenced social cognition, whereas IV administered OT, which similarly increased plasma OT concentration, did not. The data from this study supported a direct nose-to-brain effect, independent of blood absorption, for low-dose (8 IU) OT [78]. 

## 4. Drugs for GBM Treatment Administered Intranasally

Several studies have been conducted to determine the best treatment of GBM via the intranasal approach, while using monotherapy or drug combinations, including natural and/or synthetic compounds. The studies that were conducted with this purpose of developing an effective way to treat this aggressive brain tumor are summarized below.

Natural compounds, such as curcumin (CC), a polyphenolic extracted from the rhizomes of the *Curcuma longa* that presents anti-oxidant and anti-inflammatory characteristics, are interesting in the treatment of cancer and neurodegenerative disorders and they have been proposed for the treatment of GBM. The anticancer activity of CC has been attributed to the ability of the compound to reduce the expression of E3 ubiquitin ligase NEDD4, a neuronal precursor that is responsible for substrate recognition implicated in cancer development, and the inhibition of Notch1 and pAKT cancer cells signaling pathways, leading to glioma cell growth inhibition, apoptosis, and the suppression of migration and invasion [79,80]. Mukherjee and collaborators [81] used the intranasal route to deliver CC coupled to a glioblastoma specific antibody (CD68 Ab). The targeted CC-CD68 Ab conjugate was intranasally administered to mice in which glioma GL261 cells were implanted in the brain. Ten days after GL261 cells implantation, male adults C57BL/6 mice had CC-CD68 Ab solution in PBS intranasally administered every 72 h, while another group of animals received a solution of a commercially available lipid-complexed form of CC, i.e., Curcumin Phytosome (CCP), by intraperitoneal injection every 72 h. The intranasal delivery of CC-CD68 Ab conjugate and the intraperitoneal injection of CCP both caused GL261 brain tumor remission in 50% of mice, which confirmed that the CD68 Ab could be delivered to the brain via the intranasal route and confirming that CD68 Ab presented a targeted therapeutic effect after intranasal delivery. Furthermore, 70% of the animals that received CC-CD68 Ab intranasal and 60% of those treated intraperitoneal with CCP were still alive at day 90, while all of the control group animals, i.e., vehicle-treated mice, were already dead at that time. In the same study, a marked induction and activation of microglial NF-kB and STAT1 transcription factors were also observed. The transcription factors function together to cause the induction of nitric oxide synthase (iNOS) and, consequently, tumor regression. Therefore, the findings in this study indicate that intranasally delivered CC targeted conjugates can directly kill GBM cells and also repolarize tumor-associated microglial cells (TAMs) to the tumoricidal state [81].

Another natural compound, anthranoid 4,5-dihydroxyanthraquinone-2-carboxylic acid, which is also known as rhein, exhibits anti-inflammatory, anti-oxidant, anti-fibrosis, neuroprotective, and anti-tumor activities [82,83]. The anti-tumor activity of rhein is attributed to the inhibition of MAPK, PI3K-AKT, and HIF-1 signaling pathways and the down-regulation of VEGF signaling pathway [83,84]. Blacher and colleagues [85], aiming at demonstrating that the inhibition of the ectoenzyme CD38 in tumor microenvironment can attenuate glioma progression, conducted a study while using a syngeneic mouse glioma progression model. The animals, C57BL/6J wild-type (WT) and CD38-deficient C57BL/6J (CD38-/-) mice, were pretreated with vehicle or rhein by nasal administration. After 24 h, the glioma cells (GL261) were intracranially injected into the brains of the mice and the administration of vehicle or rhein was carried three times per week over 22 days. The researchers found that the rhein is capable of inhibiting the CD38 enzymatic activity, which reduces the microglia activation that support the progression of the tumor. In fact, the intranasal administration of rhein to WT mice significantly inhibited glioma progression, suggesting that CD38 is a therapeutic target in the tumor microenvironment and that small-molecule inhibitors of CD38 may serve as a useful approach for treating glioma. Furthermore, computed tomography (CT) images of the mice brains showed that the WT and Cd38-/- mice treated intranasally with rhein had the volume of the tumor reduced. However, this effect was significantly higher in WT mice when compared to Cd38-/- (reduction of 74 and 19% on day 22, respectively), demonstrating that rhein inhibits glioma progression and that this effect is mainly CD38 dependent. With this study, it was possible to conclude that the intranasal administration is an effective drug delivery route to the CNS and that the rhein has therapeutic potential for treating glioblastoma [85]. These data additionally support the possibility of brain access from the nasal cavity and demonstrate that the compounds can be directed to the CNS to be effective in the treatment of GBM, even in monotherapy. 

Nevertheless, other studies explored the use of compounds in combination. The study that was performed by Shingaki and coworkers evaluated the direct brain uptake from the nasal cavity of a model drug, 5-fluorouracil (5-FU), and whether the inhibition of CSF secretion by choroid plexus could lead to increased drug concentration in the brain [86]. 5-FU is a fluoropyrimidine that is widely used in the treatment of malignant tumors, such as breast, skin, colorectal, and neck [87]. This uracil pyrimidine analog is an antimetabolite drug that can inhibit the thymidylate synthase enzyme and perform a mis-incorporation of fluoronucleotides into RNA and DNA, which leads to cytotoxicity and cell death [88,89,90]. In this study, 5-FU was intravenously infused or nasally perfused in the presence and absence of intravenous administration of acetazolamide (AZA) in male Wistar rats. In groups of co-treatment, AZA (25 mg/kg) was injected 15 min. before starting the nasal perfusion of 5-FU. AZA is an inhibitor of the secretion of CSF by choroid plexus epithelial cells. In these cells, the CSF secretion is linked to the active transport of Na^+^ ions and AZA significantly decreases the activity of the Na/K ATPase [91]. The study demonstrated that the intravenous administration of AZA was able to enhance the CSF concentration of nasally administered 5-FU by 200–300% when compared to that obtained by 5-FU nasal perfusion without pre-treatment with AZA. AZA enhancement of nose-to-brain drug transport was obtained by decreasing the CSF secretion from the choroid plexus and, thus, sustaining the concentration of the nasally applied drug in the CSF [86]. These results demonstrated that 5-FU is capable of accessing the brain through nasal administration. It was concluded that the co-administration of active compounds to treat neurological diseases with drugs that can decrease CSF secretion from the choroid plexus could be an interesting alternative to the treatment of diseases into the brain, like GBM, since an enhancement of the concentrations of the active compounds in the brain is obtained. 

In another study, the same group reported a similar effect on the nasal administration of methotrexate (MTX) in male Wistar rats [92]. MTX is a folic acid antagonist that inhibits enzyme dihydrofolate reductase, having a therapeutic effect on a wide range of cancer types [93]. MTX presents a low penetration across the BBB, which limits its therapeutic use for GBM treatment by the oral route [94]. In the study, MTX was nasally administrated while using sodium carboxymethyl cellulose (CMC) to enhance the nasal residence time of the formulation, and AZA was orally administrated 30 min. before the nasal administration of MTX as a co-therapy. The brain uptakes of tritium labelled MTX after intranasal administration of a formulation containing CMC and after intraperitoneal administration were evaluated by blood samples and analysis of the cerebral cortex. MTX was administered for five days with the interval of two days between each treatment to evaluate the results after repeated administrations. The results showed that the amount of MTX quantified in CSF was higher than in the plasma 15 min. after intranasal administration, which indicated significant direct transport of MTX from the nasal cavity to the CSF. In contrast, a higher concentration was obtained in plasma when compared to those obtained in the CSF after intraperitoneal administration. At the same time, the effect of oral administration of AZA 30 min. before the nasal administration of MTX was evaluated and it was found that the co-treatment increased the concentration of MTX in CSF by 195% [92]. The study demonstrated that the intranasal administration is a promising route of the administration of drugs directed towards brain diseases and that AZA can enhance the amount of MTX in the CSF, which is in agreement with the results that were obtained with 5-FU [86,92].

In another study, MTX was loaded in chitosan microspheres that were designed for nasal administration. The microspheres were produced by spray-drying technique while using chitosan with different molecular weights to promote the nose-to-brain delivery of the MTX. The animals received MTX by the intravenous injection or MTX was intranasally administered while using a drug solution or MTX-loaded chitosan microspheres. The study demonstrated a higher concentration of MTX in rat brain tissues after intranasal administration of the MTX-loaded chitosan microspheres when compared to the MTX solution, while MTX could not be detected in rat brain sections after the intravenous administration. The fact that MTX-loaded chitosan microspheres showed a higher nose-to-brain transport, when compared to MTX aqueous solution after nasal administration, was attributed to the presence of chitosan. Indeed, chitosan is considered to be a safe mucoadhesive polymer that could effectively improve the nose-to-brain transport of a hydrophilic drug, like MTX, through intranasal administration [95].

Another study proposed the nasal delivery of temozolomide (TMZ) [96]. TMZ is efficiently absorbed after oral administration and it is available in capsules. Additionally, TMZ has shown good penetration across the BBB and a tolerable toxicity profile [97]. However, the increase in survival for the multimodal treatment with TMZ and radiotherapy is only 2.5 months when compared with radiotherapy alone and existing studies suggest that 60–75% of patients with GBM present no clinical benefit from treatment with TMZ [98]. Based on these data, a rat model bearing orthotopic C6 glioma xenografts was used to study the therapeutic effect of the intranasal administration of TMZ to exploit the brain-targeting properties of this delivery route. In fact, the intranasal administration of TMZ was proposed to limit the systemic exposure to the drug and thus reduce the toxic effects on healthy organs. The animals were treated with saline solution or with TMZ by three different administration routes, intravenous, oral, or intranasal, and the tumor size, rat survival time, and pathological changes were observed during the 40 days of the experiment. Magnetic resonance imaging showed a significant reduction in the volume of glioma xenografts in the intranasal TMZ group when compared to all of the other groups, including controls (*p* < 0.05). Analysis of proliferating cell nuclear antigen (PCNA) and tumor cell apoptosis that were obtained by immunohistochemistry and terminal deoxynucleotidyl transferase dUTP nick end labelling (TUNEL) assay demonstrated that the animals that were treated by the intranasal route presented the lowest expression of PCNA and the highest tumor cell apoptosis rate. The median survival time of the C6 glioma-bearing rats was also significantly longer in the intranasal TMZ group when compared to the other three groups. The control animals that were treated with saline solution survived 20 days, the animals treated with TMZ orally administered survived 21.5 days, animals receiving TMZ intravenously survived 19 days, while animals that were treated with TMZ intranasally survived 31 days [96]. The results presented in this study suggest that the intranasal TMZ administration can suppress the growth of C6 glioma in vivo and it might serve as an effective strategy for glioma treatment.

Intranasal administration in nude mice xenograft models carrying human glioblastoma tumors generated from the human glioma stem cell lines TG16, TG1N, and TG20 by Pineda and co-authors also tested a solution of TMZ in dimethyl sulfoxide (DMSO) [99]. The human glioma cell lines TG16, TG1N, and TG20 were administered by intrastriatal injection to ten-week-old female Swiss nu/nu mice. One month after the graft, the anesthetized mice intranasally received 10 μL of TMZ or vehicle; this procedure was repeated three times a week for two weeks. The TMZ administered intranasally delayed tumor growth and significantly extended the lifespan of the mice engrafted with TG16 and TG1N cells, but presented no effects on the tumors generated by TG20 cells that are resistant to TMZ in vitro. The presented results demonstrated that the intranasal route should be further considered as an option for TMZ delivery into the brain to treat intrastriatal brain tumors [99]. 

The studies that are reviewed above collectively demonstrate that the intranasal administration of anticancer drugs can induce benefits in the treatment of GBM and the intranasal route of administration might allow for direct access of the drugs to the brain serving as an effective strategy for glioblastoma treatment. However, meaningful comparative studies between intranasal and other administration routes (oral or parenteral) should be always duly conducted to conclusively highlight the potential clinical benefits of using the nose-to-brain delivery over more traditional but well-established administration routes.

### Clinical Trials on the Use of Intranasal Perillyl Alcohol for Glioblastoma Treatment 

Perillyl alcohol (POH) is a natural compound that belongs to the group of hydroxylated monoterpenes found in many essential oils (peppermint, spearmint, cherries, and others) [100]. The amphipathic character of POH makes it readily soluble in biological membranes and it has been found to be capable of interacting with the lipid bilayers of gliomas cells, leading to effective POH delivery into these cells [101]. A post translational Ras inhibition effect has been suggested by some studies as the main mechanism of anticancer action of POH but not observed in others. Thus, the action of POH is often described as pleiotropic, affecting multiple cell growth regulation processes [102]. 

Thirteen clinical studies were conducted while using orally delivered POH to cancer patients (ovarian, prostate, breast, colorectal, and pancreatic cancers) to establish the safety and efficacy of this molecule [103]. POH was dosed in capsules along with soybean oil, and the dose regimen included dozens of capsules per day per patient. However, no significant therapeutic response was observed, and the trials were halted before reaching Phase 3 clinical trials. In subsequent studies, the focus was shifted to the use of the intranasal route for the delivery for POH. Here we will briefly summarize the results of the clinical trials that were conducted on GBM patients, although an excellent review has been recently published on this specific topic [102].

To date, POH is the only therapeutic agent that is intended for cancer treatment employing the intranasal route that reached clinical trials Phases 1 and 2. It should be noted that these studies use an inhalation protocol, which might not solely involve the nose-to-brain delivery mechanism. Clinical trials have consistently showed the safety and tolerability of POH administered by the nasal route for up to eight years in addition to positive therapeutic responses in some cases [102,104,105]. The first clinical trial that was carried out in Brazil enrolled 37 patients with recurrent malignant glioma, including 29 with glioblastoma aging from 38 to 62 years old. POH was administered by inhalation four times a day at a concentration of 0.3% *v*/*v* to deliver a total daily dose of 220 mg. After six months, 14 patients with GBM showed partial response (one patient) or stable disease parameters (13 patients), suggesting some antitumor activity for POH [106]. A following study included 141 patients with recurrent glioblastoma divided into a treatment group, which included 83 patients with recurrent primary GBM and six with secondary GBM receiving POH and a control group with 52 patients receiving supportive care. The treatment consisted of the inhalation of POH four times per day to reach a total daily dose of 440 mg. The results showed a significant increase in survival between the POH treated groups over control group, between patients with secondary GBM over patients with primary GBM and between patients with tumor with deeper localization (thalamus, basal ganglia) over those with tumor at the lobar region. Subsequently, a four-year study with a cohort of 198 patients with recurrent malignant glioma (151 with primary GBM and 38 with secondary GBM) was conducted while again using a protocol of inhalation of POH four times a day, but adopting a higher dosing compared to previous studies (533.6 mg/day). Patients with secondary GBM had a significant increase in survival time when compared to patients with primary GBM, confirming the results of the previous studies, but, most importantly, 19% of patients that were enrolled in this trial remained in clinical remission after four years under exclusive POH inhalation treatment [104]. Santos and colleagues recently reported a study that combined the inhalation of POH (55 mg four times per day) with a ketogenic diet (KD) for three months. In the context of cancer therapy, some authors argue that the KD is viewed as a metabolic therapy and it consists of a high-fat, low-carbohydrate with adequate amounts of protein, promoting a specific metabolic state that is characterized by increased ketone body levels and low glucose levels in the blood [102]. The data showed that the 88% of patients that followed this treatment showed partial responses and stable disease parameters at the end of the study [107].

Encouraged by the positive results that were observed in the clinical trials performed in Brazil, a synthetic GMP grade POH (NEO100) is now under Phase 1/2A clinical trials in the U.S.A. sponsored by Neonc Technologies, Inc. (NCT02704858) [103]. These studies started in 2016 and are still recruiting patients with recurrent glioblastoma. The treatment protocol will follow that adopted in previous trials, with a regimen of POH being inhaled four times a day over a period of six months. Four dosing levels will be studied: 96 mg, 144 mg, 192 mg, and 288 mg per inhalation in order to determine the maximum tolerated dose (MTD). A total of 25 patients will be treated at the MTD and pharmacokinetic studies will be conducted during Phase 1 at the first dosing and after the first dose of the third cycle [103]. The study is expected to be concluded in October 2020 and no partial results were made available to date.

## 5. Drug Delivery Systems for Nose-to-Brain Delivery in Glioblastoma Therapy

Several therapies that apply novel drug delivery systems are under investigation for the treatment of GBM. Recently, nanoparticles (NP) have received significant attention due to the several advantages that they offer over conventional therapy, such as, for example, their ability in some cases to carry drugs across the BBB [34,108]. Furthermore, these systems offer controlled drug release, which potentially allow for a decrease in the frequency of administrations [109]. Moreover, nanoparticles are expected to improve the drug physicochemical stability and increase the biological availability [110,111].

The application of NP for the direct enhancement of drug delivery from the nasal cavity to the brain demonstrates great potential. The encapsulation of drugs into NP can overcome some of the problems related to intranasal administration (e.g., the poor capacity of penetration through the nasal mucosa, the rapid mucociliary clearance, and the enzymatic degradation), and thus enhance nose-to-brain drug delivery [112]. The small diameter of the NP also allows for them to be more effectively transcellularly transported to the brain [61]. Besides, NP may offer improved drug delivery to the brain, since they can prevent extracellular transport by P-glycoprotein (P-gp) efflux proteins localized in the olfactory epithelium and the endothelial cells that surround the olfactory bulb [113,114]. Additionally, the nanocarriers may also have their surface functionalized with specific ligands to transport agents even more effectively through the BBB [115]. Chitosan nanoparticles, polymeric nanoparticles, liposomes, solid lipid nanoparticles, nanoemulsions, micelles, and nanoplexes, among others, are the NP that have been mainly studied for nose-to-brain delivery. Table 1 summarizes the main features of the NP specifically designed for GBM therapy by the intranasal route in recent years and under pre-clinical stages of development.

### 5.1. Polymer-based NP

Polymeric NP are an extremely versatile formulation approach and have demonstrated great potential in drug delivery. Recently, some authors proposed melatonin-loaded poly(ε-caprolactone) (PCL) nanoparticles (MLT-NP) for intranasal administration in the treatment of GBM [119]. Melatonin (MLT) is an indolic hormone that is synthesized and secreted by the pineal gland, acting in the regulation of the circadian cycle [133]. Synthetic MLT is marketed as a dietary supplement. Therefore, MLT is not officially approved by the FDA for any specific therapeutic indication. However, there are several studies that show its action as antioxidant, antitumor, immune system modulator, and neuroprotective agent [134,135]. However, its short half-life, low oral bioavailability, poor solubility, and extensive first-pass metabolism that limit the drug’s ability to reach therapeutic concentrations limit its therapeutic use [136]. MLT-NP were characterized by an average size of 166.7 ± 6.3 nm and 51% encapsulation efficiency (EE), and showed a controlled release of MLT (71.2% release in 48 h). The formulation demonstrated strong activity against the U-87 MG glioblastoma cell line, which resulted in an IC_50_ ~2500-fold lower than that of the free MLT. Moreover, selective cytotoxicity effects of MLT-NP towards the tumor cell line were demonstrated, since, at low doses of MLT-NP, no cytotoxic effect was observed against MRC-5 human pulmonary fibroblasts. Fluorescence tomography images evidenced a rapid and direct translocation of the NP from the nasal cavity to the brain after the nasal administration of MLT-NP to rats. The in vivo pharmacokinetic study was conducted on male Wistar rat and the result showed a significant increase in the brain uptake of the MLT when MLT-NP were administered. Moreover, 0.5 h after administration, the percentage of administered MLT-NP in the brain was ~9- and ~18-fold higher than that of obtained using an MLT suspension administered intranasally and orally, respectively [119].

In a similar study, another group developed a polymeric NP formulation of carboplatin while using the biodegradable polymer poly(ε-caprolactone) [129]. Carboplatin (CPt) is an antineoplastic drug that belongs to the class of platinum-based alkylating agents and is approved for the treatment of various forms of cancer. However, the development of resistance, systemic toxicity, and rapid blood clearance are common problems that are related to CPt use in clinical practice [137]. CPt is available as a solution (Paraplatin^®^, Bristol-Meyers Squibb) for intravenous administration. Polyvinyl alcohol (PVA) was selected as the emulsifying agent for the production of the polymeric NP, as it provides the nanocarriers with lower particle size and enhances the maximal EE, while at the same time reducing particle aggregation. The in vitro drug release studies showed that the drug was released from the NP with a biphasic pattern characterized by an initial burst, followed by a prolonged sustained release due to a non-Fickian diffusion. Permeation studies across sheep nasal mucosa provided data similar to the in vitro release studies. In vitro cytotoxicity towards LN229 human GBM cells showed enhanced cytotoxicity by CPt-loaded NP only for long incubation times (96 h). In situ nasal perfusion studies that were conducted in Wistar rats with two CPt-loaded PCL NP containing different amounts of PVA demonstrated that both of the formulations showed progressive nasal absorption of CPt over time. Indeed, nanoencapsulated CPt showed better nasal absorption when compared to the free drug, as indicated by the smaller amount of CPt detected in the perfusate after intranasal administration [129].

GBM is characterized as a highly vascularized tumor as a consequence of the overexpression of endothelial growth factor (VEGF) [138]. Bevacizumab (BCZ; Avastin^®^, Roche), an anti-VEGF monoclonal antibody, was approved by the FDA (2009) to be used as a single agent in the treatment of patients with recurrent GBM [139]. However, BCZ failed to improve overall patient survival rates in some clinical studies [140,141]. Sousa and collaborators developed BCZ-loaded PLGA NP with the objective of improving BCZ bioavailability and targeting the drug efficiently to the brain [116]. BCZ-loaded PLGA NP (185.0 ± 3.0 nm with slightly negative charge) and control (Avastin^®^) were intranasally administered to CD-1 mice for seven days. The in vivo pharmacokinetic study demonstrated a significant increase of BCZ concentration in the brain, with 5400 ± 2313 ng/g brain tissues and 1346 ± 391 ng/g brain tissues for PLGA NP and control, respectively. The amount of BCZ that was found in blood and off-target organs (lung and liver) was insignificant in the case of PLGA NP formulation. On the other hand, BCZ was found in the blood, lung, and liver after intranasal administration of Avastin^®^ control. Thus, the improvement offered by nose-to-brain delivery might be an alternative for decreasing the systemic side effects of BCZ. The authors examined the efficacy of the nanoformulation in a nude mice orthotopic GBM model while using bioluminescence and VEGF quantification. Upon nasal administration once a week for 14 days, the BCZ-loaded PLGA NP showed a significantly higher anti-angiogenic effect as compared to Avastin^®^ intranasally administered. However, in terms of tumor growth, no statistical difference was observed between the treatments. The authors attributed these results to the previously reported slow release profile of BCZ from PLGA NP [142]. They also suggested that another in vivo model should be used to increase the number of administrations and evaluate tumor growth for a longer time [116].

**Figure 3:**
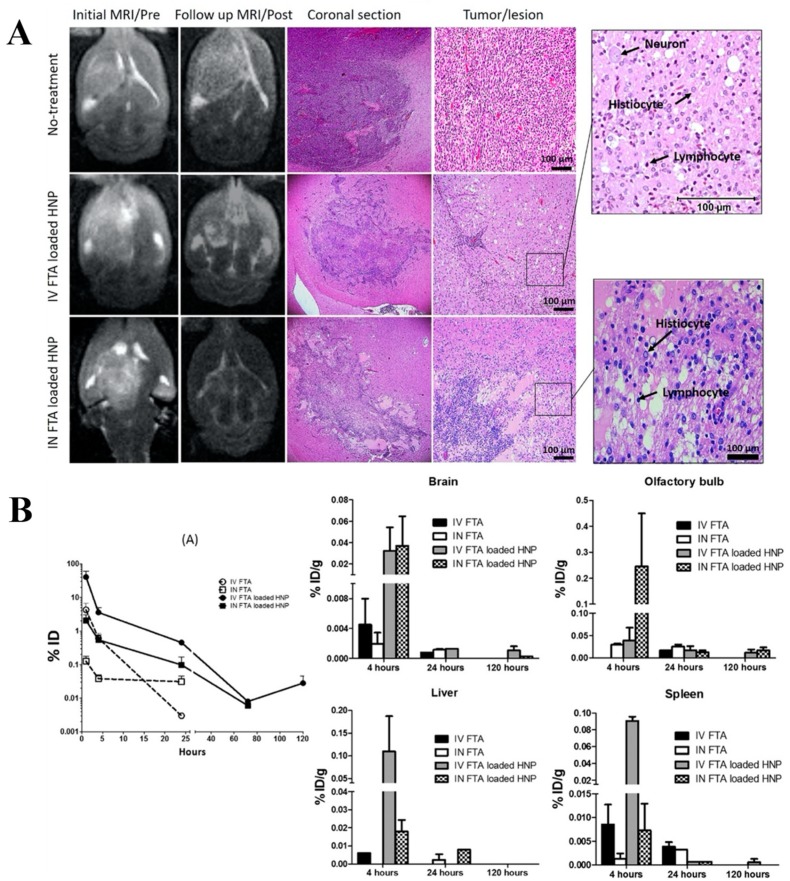
Initial/pre-treatment and follow up/post-treatment MRI images of rat brains from non-treated animals or after treatments with IV or IN farnesylthiosalicylic acid (FTA)-loaded hybrid nanoparticles (HNP) formulations and their corresponding coronal brain sections stained with hematoxylin and eosin (Panel A). In the coronal brain sections, the upper panels show a dense tumor area in the right *striatum* of non-treated rats, whereas the middle and lower panels show cellular re-organization of tumor cells after treatment with IV or IN administered FTA-loaded HNP, respectively. Presence of inflammatory response is shown by the abundant presence of histiocytes and lymphocytes. Biodistribution study of the formulations in healthy rats (Panel B). (**A**) Plasma FTA concentration *versus* time profile for the four treatment formulations. (**B**) Distribution of FTA in brain, olfactory bulb, liverm and spleen of healthy rats after 4, 24, and 120 h post-administration (reproduced with permission from [123]).

Another group worked on lipid-PEG-PLGA hybrid nanoparticles (HNP) for intranasal delivery of farnesylthiosalicylic acid (FTA) with the aim of increasing the brain-targeting efficacy [123]. FTA, which is also known as salirasib, is a synthetic derivative of salicylic acid. FTA is a potent and specific inhibitor of Ras proteins, which are found in most malignant tumors [143]. However, FTA presents poor oral bioavailability and it is not able to cross the BBB at effective concentrations [144]. HNP were produced by the emulsion sonication method and it showed a particle size of around 160 nm and negative surface charge (−12 mV). The in vitro cytotoxicity after 24 h showed that the hybrid nanocarriers significantly decreased rat glioma-2 (RG2) cells viability of ~60%, as compared to only ~13% obtained while using free FTA treatment. Furthermore, cytotoxicity studies towards healthy cells evaluated using L929 mouse fibroblasts evidenced a significant toxic effect for free drug treatment, whereas FTA-loaded HNP did not show significant toxicity. For the in vivo studies, the RG2 cells were unilaterally implanted into the right *striatum* of female Wistar rats. After 10 days, glioma bearing rats received a single dose intravenous treatment or five repeated doses intranasally of FTA-loaded HNP (500 μM/20 μL). Free FTA and blank HNP were used as controls. The data obtained by magnetic resonance imaging (MRI) showed that tumor area decreased by 57.3% and 31.0% when compared to the controls for the single intravenous and repeated intranasal doses of HNP, respectively. In fact, intravenous and intranasal administrations of free drug and blank nanocarriers both had no significant effect in vivo. After a treatment period of five days, the intranasal administration of the nanocarrier achieved a significant decrease of 55.7% in tumor area, which was similar to that observed by intravenous administration of the same formulation (Figure 3A). This result was corroborated by the in vivo distribution studies that indicated that the percentage of the FTA dose reaching the brain was similar after intranasal and intravenous administration of HNP (Figure 3B). However, the highest accumulation of NP was detected in the olfactory bulb after intranasal administration, whereas following intravenous administration the nanocarrier caused a high accumulation of FTA in the spleen and liver (Figure 3B) [123].

Recently, several researchers have proposed the inclusion of nanoformulations within mucoadhesive gelling systems for nasal administration to enhance the nasal residence time and reduce the mucociliary clearance [145,146]. For example, Jain and collaborators developed an innovative MTX formulation for GBM by encapsulating the drug into polymeric PLA NP (MTX-NP) and including poloxamer 188 in combination with Carbopol 934 in the formulation to obtain a thermosensitive hydrogel [128]. It was demonstrated that the MTX-NP formulation mucoadhesion correlates with the amount of Carbopol 934 included while using a mucoadhesiveness testing apparatus. In vivo studies that were carried out using male Wistar rats indicated that the combination of the in situ gelling system and NP resulted in an increase of MTX in the brain when compared to data that were obtained with the MTX solution. The pharmacokinetic parameters demonstrated an increase in area under the plasma concentration–time curve (AUC) for the drug when administered through the nasal route when compared to the administration through the intravenous route. Moreover, PLA MTX-NP enhanced the maximum drug concentration (C_max_) and AUC 1.5 times as compared to the control MTX solution that was administered by the nasal route [128].

### 5.2. Lipid-Based NP

The encapsulation of drugs into nanocarriers has enhanced the therapeutic potential of a wide variety of molecules in view of the treatment of GBM. Among several bioactive compounds, many researchers have shown interest in the nanoencapsulation of curcumin. In fact, NP are able to overcome a number of limitations that are related to this natural compound, such as low solubility, low oral bioavailability, and low capacity to cross the BBB [147,148].

In this context, Madane and Mahajan developed a nanostructured lipid carrier (NLC) system for curcumin (CC) while using hot high-pressure homogenization [124]. CC showed a biphasic release pattern from NLC formulations, initially showing a burst release of approximately 25%, followed by a sustained release up to 24 h. Moreover, an ex vivo permeability study that was carried out using Franz diffusion cells showed greater drug permeability across sheep nasal mucosa of CC that were formulated in the NLC system when compared to the free drug suspension. The in vitro cytotoxicity studies using the human astrocytoma-glioblastoma cell line U-373 MG showed IC_50_ values of 9.8 ng/mL for the nanoformulation and 13.6 ng/mL for the positive control (adrenomycin), demonstrating the potential effectiveness of CC-NLCs against glioblastoma. Biodistribution studies in Wistar rats showed a higher drug concentration in the animal brain after intranasal administration of CC-NLCs than free drug suspension (C_max_ was 5.4321 ± 2.098 ng/g with t_max_ 180 min. and 8.6201 ± 8.182 ng/g with t_max_ 120 min. for the free drug and for CC-loaded NLC, respectively) [124].

In another study, Shinde et al. investigated the brain bioavailability and efficacy in vitro of CC-loaded microemulsions (ME) after nasal and intravenous administration in rats [125]. The proposed drug delivery system consisted of microemulsion formulated with CC and docosahexaenoic acid (DHA), which, in addition to improving the CC bioavailability, also has antitumor effects of its own. In fact, the results of in vitro cytotoxicity studies showed a synergistic effect of CC with DHA that was formulated in a ME against the human U-87 MG glioblastoma cell line. Indeed, the IC_50_ value was 3.7 ± 0.2 ng/mL for CC-loaded DHA-ME, 502.7 ± 24.6 ng/mL for CC-ME, while it was 747.8 ± 53.0 ng/mL for a simple CC solution, thus confirming the synergistic anticancer effect of CC and DHA in the ME. It was suggested by the authors that the anticancer activity of DHA could be due to its natural affinity to the neuronal cells and DHA capacity to induce lipid peroxidation. Moreover, the combination of CC with DHA and its subsequent encapsulation in ME increased the distribution to the brain after intravenous and intranasal administration in healthy rats. CC brain concentrations following intranasal administration were strikingly higher when compared to intravenous administration, especially in the case of the MEs. In particular, the brain targeting efficiency (DTE) and direct transport percentage (DTP) that were calculated for the CC-loaded DHA-ME were 1,615% and 97%, respectively [125].

On a similar note, Gadhave and his team worked on ME and mucoadhesive hydrogel (MME) for the intranasal delivery of teriflunomide (TFM) with the aim of increasing the brain delivery of TFM [118]. TFM is a selective and reversible inhibitor of the mitochondrial enzyme dihydroorotate dehydrogenase that is necessary for the de novo synthesis of pyrimidine nucleotides [149]. The FDA approved the TFM in September 2012 for the treatment of adults with multiple sclerosis and it is available as a tablet for oral administration (Aubagio^®^, Sanofi-Aventis). However, it has been reported that the oral administration of TFM should be performed with caution due to the high risk of severe liver injury [150]. Recent studies have demonstrated the potential of TFM as an antitumor agent in breast cancer [151], prostate cancer [152], and lung cancer [153]. The development and optimization of TFM-MME were performed while using a Box-Behnken design of experiments. The optimized formulations were formulated by using the mixture of mucoadhesive agents HPMC K4M (0.3%) and Poloxamer 407 (17%). In the cytotoxicity assay that was carried out in the human U-87 MG glioblastoma cell line, the authors used carmustine as positive control. After 48 h of treatment, the cell viability was reduced to 38.5% and 37.8% at 160 μg/mL for carmustine and TFM-MME, respectively, which indicated that the cytotoxicity profiles against glioma cells were comparable. The in vivo biodistribution study in Swiss Albino mice was assessed by gamma scintigraphy via ^99m^Tc labeling of the particles. The TFM-MME formulation showed enhanced brain accumulation (C_max_ 0.62% RA/g) with a DTPof 99.2% and a DTE of 359% when compared with the intravenous administration of TFM-ME. However, the in vitro and in vivo studies did not include the free TFM controls as comparators with the proposed ME. The in vivo safety of TFM-ME and TFM-MME was evaluated in toxicological studies that were carried out while using male Wistar rats receiving daily administrations of the two formulations for 28 days. TFM-MME formulation did not reflect any changes in liver or kidney biomarkers, hematology, and histopathological examination at low and medium doses. The study still needs more robust in vitro and in vivo investigations to demonstrate the efficacy of the TFM-ME and TFM-MME for treatment of GBM, although these formulations were demonstrated to be safe for nasal administration [118].

Colombo and collaborators investigated the brain biodistribution and antitumor efficacy of nanoemulsions containing kaempferol (KPF) that were prepared by high-pressure homogenization with and without chitosan [121]. KPF is a natural flavonol that is found in several species of edible plants (berries, broccoli, apples, grapes, cabbage, and beans) and medicinal plants (*Ginkgo biloba*, *Rosmarinus officinalis*, *Aloe vera*, *Centella asiatica*, and *Hypericum perforatum*) [154]. This compound has shown antioxidant, anti-inflammatory, and anti-tumor activities [155]. Despite its excellent properties, it is a substance with low aqueous solubility and low oral bioavailability [156]. As a consequence, the FDA does not approve KPF and there are no pharmaceutical formulations that are available in the market containing this natural compound. However, when formulated in a nanoemulsion coated with chitosan, the amount of KPF permeating across pig nasal mucosa in ex vivo diffusion studies while using Franz diffusion cells significantly increased. Furthermore, during in vitro experiments, the formulation that was coated with chitosan reduced C6 glioma cell viability through the induction of apoptosis to a greater extent than either unencapsulated KPF or a chitosan-free nanoemulsion loaded with KPF. The IC_50_ values of the formulation that was coated with chitosan was about 20-fold smaller than free KPF. In vivo studies in Wistar rats indicated a significant increase in brain uptake after intranasal administration in comparison to the control KPF solution. The formulation that was coated with chitosan significantly enhanced the amount of drug reaching the brain. The KPF brain concentration that was detected after nasal administration of chitosan coated KPF-loaded nanoemulsion was, in fact, 5- and 4.5-fold higher than that obtained using the free drug solution and KPF-loaded nanoemulsion without chitosan, respectively. The increased KPF concentration in the brain was not only attributed to the intranasal administration, but also to the mucoadhesive properties and efficient permeation enhancement provided by chitosan [157,158].

Khan and collaborators proposed another approach based on lipid nanostructures [122], who developed nano-lipid chitosan hydrogel formulations for the nose-to-brain delivery of TMZ. The formulations were prepared by high-pressure homogenization while using vitamin E, as the lipid and Gelucire^®^ 44/14, polysorbate 80, and Transcutol^®^ as surfactants. Afterwards, the nano-lipid formulations were dispersed in chitosan (1.0% *w*/*v*) and converted to a hydrogel. The optimized formulation (size 134 nm, −13.11 mV, EE% 88.45) was able to control the TMZ release (60% release in 24 h) and increase the TMZ permeability of the TMZ by 2.5 times across nasal mucosa. Along with this, the formulation increased the in vitro cytotoxicity of TMZ towards the C6 glioma cell line. In vivo studies in Wistar rats showed an increased DTE of 326% and a DTP of 93% after intranasal administration of chitosan gel formulation containing the TMZ-loaded nano-lipids in comparison to the intranasal administration of nano-lipid formulation as a control (DTE, 113.36% and DTP, 71.74%) [122].

Recently, Azambuja and co-workers developed cationic nanoemulsions (NE) to deliver CD73 small interfering RNA (siRNA CD73) for GBM treatment through the intranasal route [117]. Ecto-5′-nucleotidase (CD73) regulates the extracellular adenosine monophosphate (AMP) and adenosine levels, which have been described as proliferation and drug resistance factors [159,160]. Moreover, CD73 is overexpressed in GBM cells and its inhibition impairs tumor progression [161]. The cationic nanoemulsions were manufactured by microfluidization while using lecithin, medium chain triglycerides, and 1,2-dioleoyl-sn-glycero-3-trimethylammonium propane (DOTAP). The NE-siRNA CD73 were prepared by the adsorption of siRNA to blank formulations (ζ-potential +32 mV) while using different theoretical ratios of cationic lipids to siRNA. In vitro studies using rat C6 glioma cells demonstrated that the NE-siRNA CD73 efficiently decrease cell viability after 48 h of treatment. On the other hand, NE-siRNA scramble that was used as control did not induce any alteration in C6 glioma cell viability. Additionally, cytotoxicity studies showed that the formulation was safe and it did not produce any toxicity in rat primary astrocyte cultures. Interestingly, it was demonstrated that tumor cells took up NE-siRNA CD73, both in vitro and in vivo, which resulted in CD73 knockdown. The in vivo results in glioblastoma-bearing rats demonstrated that NE-siRNA CD73 treatment by intranasal administration significantly decreased the glioma growth by 60% when compared to the control groups (untreated and NE-siRNA scramble). Furthermore, neither NE-siRNA CD73 nor NE-siRNA scramble treatment induced systemic toxicities to glioblastoma-implanted rats [117].

### 5.3. Polysaccharide NP

Galectin 1 (Gal-1) is a protein that is over-expressed in GBM and is highly associated with tumor progression [162]. The knockdown of Gal-1 using siRNA administration has shown promising results in GBM. Van Woensel and collaborators recently developed chitosan nanoparticles that were loaded with a Gal-1 siRNA for nasal delivery to treat GBM [126]. Gal-1 siRNA loaded chitosan NPs were spontaneously formed by direct complexation due the electric interaction of positively charged chitosan and negatively charged siRNA, which resulted in the successful encapsulation of the siRNA in the nanoparticles and protecting them from ribonucleases (RNases). The NP strongly adhered to the nasal mucosa and the siRNAs were detectable up to 8 h after administration, while the free siRNA only showed weak mucoadhesion. This was attributed to the mucoadhesive properties of chitosan that allowed the nanoparticles to overcome mucociliary clearance and improve the retention time in the nasal cavity [158]. In addition, the encapsulated siRNAs were effectively delivered to the glioma cells from the nasal cavity, since a strong reduction in Gal-1 expression was observed. There was also a reduction in the vascular diameter of the tumor microenvironment in the GL261mice brain tumor model [126].

In a subsequent study, the same group showed that Gal-1 knockdown obtained through nasal administration of chitosan nanoparticles that were loaded with a Gal-1 siRNA displays synergistic effects with TMZ oral treatment and immunotherapy with dendritic cell (DC) vaccination or programmed cell death protein-1 (PD-1) blockade via intraperitoneal administration, suggesting the possibility of combination therapy. The intranasal delivery of Gal-1 siRNA induced a remarkable switch in the tumor micro-environment cellular composition, which reduced macrophage polarization from M1 (pro-inflammatory) to M2 (anti-inflammatory) and inhibited the recruitment of monocytic myeloid derived suppressor cells during GBM progression. Furthermore, the results demonstrated that the median survival increased from 32 days in TMZ treated mice, to 53 days in mice that were orally and nasally treated with TMZ with chitosan nanoparticles loaded with a Gal-1 siRNA. The prophylactic vaccination model showed that the combining DC vaccine with chitosan nanoparticles that were loaded with a Gal-1 siRNA intranasally administered also increased the median survival to 53 days. Similarly, the concomitant intranasal administration of chitosan nanoparticles loaded with a Gal-1 siRNA improved the therapeutic effect of anti-PD-1 antibodies, and increased the median survival to 51.5 days when compared to the control groups (17.5 and 30 days for untreated mice and anti-PD-1 antibodies alone, respectively) [127].

### 5.4. Targeted NP

The functionalization of the surface of nanocarriers with targeting moieties is one strategy for improving brain tumor accumulation of drug delivery systems. Ephrin type-A receptor 3 (EPHA3) is a membrane-associated receptor that is overexpressed in the stroma and vasculature of gliomas [163]. Chu and co-authors developed PLGA NP functionalized with anti-EPHA3 antibodies for direct nose-to-brain delivery of temozolomide butyl ester (TBE) [120]. Nanoparticles that were loaded with TMZ were prepared by emulsion-solvent evaporation method and subsequently coated with N-trimethylated chitosan (TMC) and their surface functionalized with anti-EPHA3 antibodies. The drug release studies showed a sustained release of TMZ from the nanoparticles by up to 48 h. The results of a cytotoxicity assay on the C6 cells and of nanoparticles cellular uptake demonstrated that anti-EPHA3 functionalization could enhance GBM targeting increasing the cytotoxic effect of the drug. Furthermore, the fluorescence distribution and anti-glioma efficacy in glioma-bearing rats confirmed that the enhanced antitumoral effects were attributed to the nanoparticles surface modification. Anti-EPHA3 functionalized nanoparticles increased the median animal survival by 1.37-folds when compared to the non-targeted nanoparticles. Overall, the author concluded that anti-EPHA3 modified PLGA NP might potentially serve as a nose-to-brain drug carrier for the treatment of GBM [120].

Kanazawa et al. performed a comparative study between methoxy[poly(ethylene glycol)]-*b*-[poly(ε-caprolactone)] (MPEG-PCL) polymer micelles and the trans-activator of transcription (TAT) peptide-modified MPEG-PCL micelles [164]. TAT is a cell-penetrating peptide (CPP) that is derived from human immunodeficiency virus type 1 (HIV-1) containing a protein transduction domain that can induce endocytosis [165]. Polymer micelles were prepared by self-assembly exploiting the amphiphilic properties of the block copolymer. The use of micelles that were modified with TAT and loaded with a model drug, i.e., coumarin, showed an enhancement of direct intranasal brain delivery. Furthermore, the effect of particle size (100, 200, 300, and 600 nm) was investigated on brain distribution after the intranasal administration to glioma C6 cells-bearing rats. The coumarin concentrations in the brain administered with 100 nm micelles were significantly higher than in rat brain that was administered with 600 nm. Interestingly, the drug concentrations in the left side of the brain were higher than those in the right non-inoculated side [164].

Camptothecin (CPT) was encapsulated in TAT-modified micelles and administered by the intranasal route in rats in a later study from the same group. CPT, a quinolone alkaloid, is an inhibitor of the nuclear enzyme DNA-topoisomerase I, which relieves the DNA torsional strain by inducing reversible single-stranded breaks. This naturally occurring alkaloid is extracted from the bark of the Chinese tree, *Camptotheca acuminata* [166]. Even though CPT has shown interesting antitumor activity, its clinical use is limited by extremely low solubility, poor stability, and systemic toxicity. In fact, CPT was discontinued during Phase II trials in 1972 although the initial clinical trials had shown strong antitumor activity. CPT caused severe and unpredictable adverse effects, including myelosuppression, vomiting, diarrhea, and severe hemorrhagic cystic disease [167]. An in vitro cytotoxicity study in rat C6 glioma cells indicated that the CPT-loaded MPEG-PCL-TAT micelles showed higher cytotoxicity than CPT-loaded MPEG-PCL micelles. In vivo, when compared to unmodified micelles, TAT-modified micelles significantly increased the median survival time of rats bearing intracranial glioma tumors. Furthermore, body weight was significantly reduced when compared to untreated rats after seven days of nasal treatment with a simple CPT solution, which indicated severe systemic toxicity. In contrast, CPT-loaded MPEG-PCL or CPT-loaded MPEG-PCL-TAT did not cause significant changes in total body weight, which suggested that micellar formulations were effective in reducing the systemic toxicity of the drug [131].

**Figure 4:**
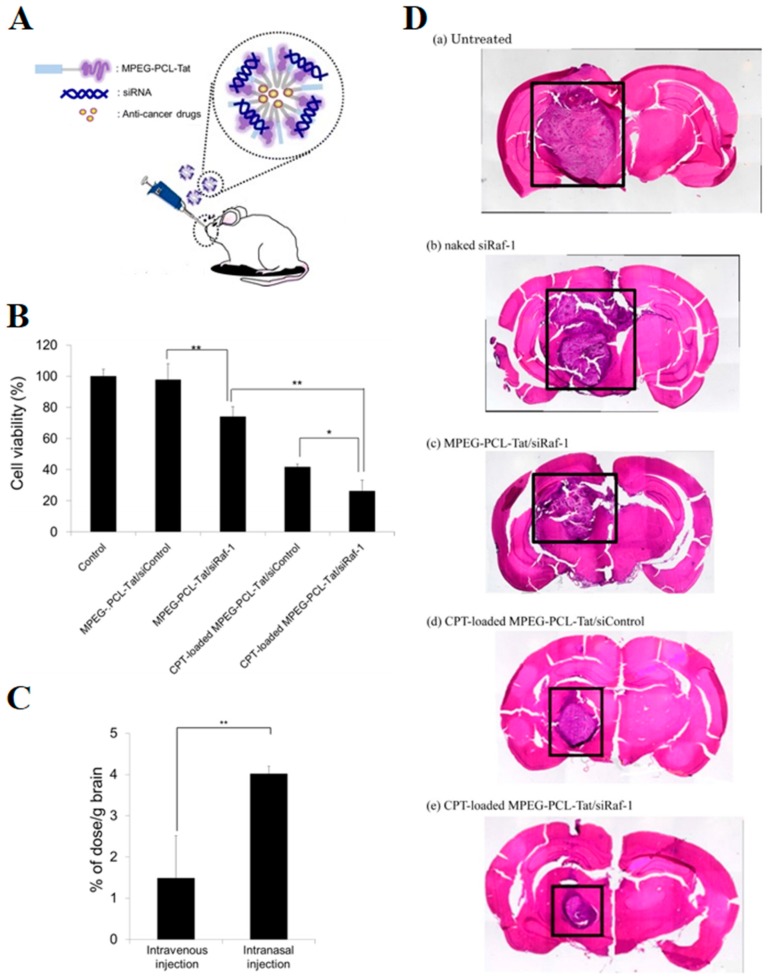
In vitro and in vivo efficacy of cell-penetrating peptide-modified micelles. (**A**) Illustrative model for camptothecin (CPT)-loaded MPEG-PCL-Tat/siRaf-1. (**B**) In vitro cytotoxicity (WST-8 assay) in C6 glioma cells transfected with CPT-loaded MPEG-PCL-Tat/siRaf-1 complexes. (**C**) Distribution of siRNA in brain tissue after intravenous or intranasal administration of MPEG-PCL-Tat/siRNA complex. Rats were killed after the administration of siRNA/MPEG-PCL-Tat complex (20 μg as siRNA), and each brain was enucleated. (**D**) Images of HE-stained brain tissue in intracranial C6 glioma-bearing rats after intranasal administration of siRaf-1 complexed with camptothecin-loaded micelles. After 2 weeks, tissues were taken from untreated rats (**a**) and rats treated with naked siRaf-1 (**b**), MPEG-PCL-Tat/siRaf-1 complex (**c**), CPT-loaded MPEG-PCL-Tat/siControl (**d**), and CPT-loaded MPEG-PCL-Tat/siRaf-1 (**e**) (* *p* < 0.05, ** *p* < 0.01) (adapted with permission from [132]. Copyright 2014 American Chemical Society).

This approach has also been studied to improve the co-administration of anticancer drugs and siRNA to the brain [132]. MPEG-PCL-TAT micelles were loaded with anti-rat Raf-1 siRNA (siRaf-1) and CPT and then evaluated for their brain uptake efficiency on a C6 glioma model (Figure 4A). When compared to intravenous delivery, intranasal delivered MPEG-PCL-TAT loaded with siRaf-1 significantly enhanced the nucleic acid concentration in rats brain (Figure 4C). As shown in Figure 4B and D, significant inhibition of tumor growth in vitro and in vivo was demonstrated. This was attributed to the combined effects of the CPT and the Raf-1 gene silencing of siRaf-1 in glioma tissues [132].

## 6. Stem Cells for Treatment of GBM

Stem cells have been proposed in recent years for glioma therapy [168,169,170,171]. These cells have a tropism for brain tumoral tissue, a minimum tropism for normal neural cells, and the capability to cross the BBB [172,173,174,175,176]. Stem cells can be derived from multipotent stem cells, such as mesenchymal stem cells (MSCs) and neuronal stem cells (NSCs) [176]. MSCs are hematopoietic stem cells and they can be isolated from different tissue sources, making them easier to isolate than NSCs [177,178]. MSCs have the ability to self-renewal, to differentiate in specific functional cellular and immune-compatible nature [176]. MSCs established from adipose tissue [179], human dermis [180], and human umbilical cordon [181] can inhibit tumor cells proliferation through the secretion of a tumor suppressor soluble molecule, the dickkopf-1 (DKK-1). The authors observed an antitumoral effect through the inhibition of glioma cells migration, invasion, proliferation, and survival in a study with normal rat embryonic NSCs. Interestingly, the levels of mutant p53, serine/threonine kinase AKT phosphorylation, and extracellular signal-regulated protein kinases (ERK1/2), all of the pathways related to GBM uncontrolled growth, evasion of apoptosis, and enhanced tumor invasion were decreased [182].

The intranasal route is considered to be an interesting approach to administer stem cells. A study of Reitz and co-workers focused on the nasal delivery of neural stem/progenitor cells (NSPCs) to target brain tumors [183]. Intracerebral human (U87 and NCE-G55T2) and murine (syngenic Gl261) glioma cell-based glioblastoma models were used to evaluate the specific accumulation in mice brain of the NSPCs. The NSPCs intranasal administration was initiated ten days after glioma cells injection. The histological analysis performed five days after the start of the treatment demonstrated the presence of NSPCs in peritumoral and intratumoral areas of brain. The absence of NSPCs in the brains of animals belonging to the control group confirmed the NSPCs tropism for the tumor. The distribution study showed that the cells entered in the brain tumor area 6 h post-administration. The migration initially occurred (within 24 h) via the olfactory pathway, while the cells migrated later via the microvasculature of nasal mucosa [183]. In a different study, it was demonstrated that the nose-to-brain migration of MSCs that were delivered into the nasal cavity occurred via the olfactory and trigeminal pathways [184].

Stem cells have also been proposed as carriers to deliver cytotoxic agents with the intent to exploit their tropism towards brain tumors. Dey and co-workers evaluated the ability of NSCs to carry the oncolytic virus (OV) CRAd-S-pK7 by intranasal administration [185]. CRAd-S-pK7 virus selectively infects tumor cells [186] and the stem cells were able to efficiently deliver OV in various models of glioma [187,188,189]. In this study, NSCs were genetically modified without changes in their phenotype to improve their tropism towards tumor signaling. In two mice models of malignant glioma (GBM43 and GBM6 intracranial xenografts), the administration of NSCs by intranasal route extended the survival of CRAd-S-pK7 viruses in glioma tissue. This result was attributed to an efficient migration of modified NSCs to the brain tissue and the successful delivery of CRAd-S-pK7 to the tumor site. In addition, the authors were able to verify an extension of animal survival when treated with NSCs carrying the OV in association with radiotherapy (median survival benefit of five days) [185].

In another study, Balyasnikova and co-workers demonstrated that MSCs expressing TNF-related apoptosis-inducing ligand (TRAIL) were able to reach the tumoral tissue and improve the median survival of the irradiated mice with intracranial U87 glioma xenografts in comparison to the non-irradiated and irradiated control mice [190]. TRAIL is an anticancer protein that is expressed and secreted by several stem cells. Additionally, it selectively promotes apoptosis in glioma cells with minimal effects on healthy cells [177]. The authors also verified the rapid MSCs delivery via the nasal cavity, with the detection of MSCs in the animal brains already within 2 h after administration and their subsequent infiltration in the intracranial tumors.

The stem cells approach for GBM treatment has also been combined with nanotechnology. Mangraviti and co-workers developed a system that combined polymeric nanoparticles and human adipose tissue derived MSCs to deliver bone morphogenetic protein 4 (BMP4) and evaluated the antitumor effect in a primary malignant glioma model [130]. Polymeric NP of poly(beta-amino ester)s (PBAE) were determined to be a good option for the transfection of MSCs due to their favorable physicochemical characteristics: hydrodynamic diameter next to 220 nm, polydispersity index lower than 0.2, and positive zeta potential. Thus, MSCs were transfected with polymeric nanoparticles to express BMP4 and subsequently administered via an intranasal route in rats. The treatment with BMP4 expressing MSCs significantly improved the survival of tumor bearing animals: 60% of treated rats survived up to 16 days after treatment with a 21.4% increase in median survival time over the control group animals [130].

## 7. Conclusions

GBM is a devastating brain disease with an extremely poor prognosis. Usually, the oral route of administration is considered to be the most convenient for patients. However, for the pharmacological treatment of GBM, it is essential for drugs to reach the brain in their bioactive form. Yet, the therapeutic agent has to overcome several biological barriers when orally administered, such as enzymatic degradation, first-pass metabolism, and BBB. At the moment, TMZ is the chemotherapy agent most used clinically in the treatment of GBM and it is orally administered. In the treatment of GBM, the intranasal route of administration and, more specifically, nose-to brain delivery, potentially presents several advantages over the oral and parenteral route for administering anticancer drugs. In this review, we reported that different therapeutic agents (small organic molecules, biotech compounds, stem cells) are under investigation for GBM treatment exploiting intranasal delivery. In terms of delivery systems, drugs that are entrapped into nanostructured carriers (nanoemulsions, microemulsions, and polymeric and lipid nanoparticles) have been designed and investigated to enhance nose-to-brain delivery. Some of these innovative formulations present surface modified nanocarriers that are able to target receptors and proteins overexpressed in GBM cells facilitating the specific drug delivery to the tumor cells. Nevertheless, most studies are currently only in a pre-clinical phase of development, where promising results remain based on animal models. Overall, the data that were obtained in pre-clinical models suggested a better biodistribution and an improved therapeutic effect of anticancer compounds after intranasal delivery. In addition, long-term clinical studies that were carried in humans with POH demonstrated patient compliance and promising results in selected GBM patients while using this route of delivery. Although there are still a restricted number of studies specifically focused on the intranasal delivery of anticancer compounds to treat GBM, this strategy has the potential to be clinically used for both innovative new drugs and drugs already in use, and may lead to new therapeutic options for GBM patients in the near future.

## Figures and Tables

**Table 1:** Characteristics and pre-clinical findings in the last 10 years using nanocarriers administered by the intranasal route for GBM therapy.

Drug	Type of Nanocarrier	Surface Modification	Preparation Method	Size (nm)	Zeta Potential (mV)	In Vivo Model	Ref.
Bevacizumab	Polymeric NPs(PLGA)	-	Emulsification-evaporation	185.0 ± 3.0	~−2	U-87-luc mice	[116]
Ecto-5′-nucleotidase (CD73)	Nanoemulsion	-	Microfluidization	262.7 ± 12.8	+3.5 ± 3.0	C6 rat glioma	[117]
Teriflunomide	Microemulsion	-	Progressive aqueous phase titration	22.81 ± 0.48	−22.62 ± 1.1	-	[118]
Melatonin	Polymeric NPs(PCL)	-	Nanoprecipitation	166.7 ± 6.3	−34.0 ± 5.2	-	[119]
Temozolomide	Polymeric NPs(PLGA)	Anti-EPHA3	Emulsion-solvent evaporation	125 to 146	−21 to + 23	C6 rat glioma	[120]
Kaempferol	Nanoemulsion	Chitosan	High-pressure homogenization	180.53 ± 4.90 (coated) 145.07 ± 4.91 (uncoated)	+26.09 ± 2.67 (coated) −18.10 ± 2.55 (uncoated)	-	[121]
Temozolomide	Nanoemulsion	-	High-pressure homogenization	134 nm	−13.11	-	[122]
Farnesylthiosalicylic acid	Hybrid nanoparticles	-	Emulsion sonication	164.3 ± 10.3	−12.0 ± 1.3	RG2 rat glioma	[123]
Curcumin	Microemulsion	-	Oil titration method	<20	~+10	-	[124]
Curcumin	Nanostructured Lipid Carriers	-	High pressure homogenization	146.8	−21.4 ± 1.87	-	[125]
siRNA	Chitosan nanoparticles	-	Ionic gelation	141 ± 5	+32	GL261 tumor bearing mice	[126]
siRNA siRNA + TMZ or immunotherapy	Chitosan nanoparticles	-	Ionic gelation	141	+32	GL261 tumor bearing mice	[127]
Methotrexate	Polymeric nanodispersion(PLA)	-	Emulsion/Solvent evaporation	351 ± 13.4	+25.1 ± 1.2	-	[128]
Carboplatin	Polymericnanoparticles(PCL)	-	Double emulsion/solvent evaporation	311.6 ± 4.7	−16.3 ± 3.7	-	[129]
BMP4 plasmid DNA	Polymeric nanoparticles(PBAE)	-	Self-assembly	218 ± 7	+17 ± 1	U87 rat glioma	[130]
Camptothecin	Polymer micelles(MPEG-PCL)	Tat	Self-assembly	88.5 ± 20.2	+10.4 ± 2.84	C6 rat glioma	[131]
siRaf-1/Camptothecin	Polymer micelles(MPEG-PCL)	Tat	Self-assembly	60 to 200	−2.86 to 15.9	C6 rat glioma	[132]

Abbreviations: PLGA, Poly(lactic-co-glycolic acid); PCL, Poly(ε-caprolactone); PLA, Poly(lactic acid); PBAE, Poly(beta-amino ester); MPEG-PCL, Methoxy[poly(ethylene glycol)]-b-[poly(ε-caprolactone)] amphiphilic block copolymers.
